# Supplementary material for: Arene-Ruthenium(II) Complexes with Carbothiamidopyrazoles as a Potential Alternative for Antibiotic Resistance in Human
Source: Molecules. 2022 Jan 12;27(2):468. doi: 10.3390/molecules27020468 (PMC8781304; doi:10.3390/molecules27020468)
Supplement: Supplementary file 1 [file molecules-27-00468-s001.zip › molecules-1527759-supplementary.pdf]

SUPPLEMENTARY MATERIAL

Arene-Ruthenium(II) Complexes with Carbothiamidopyrazoles as a Potential Alternative for Antibiotic Resistance in Human

Ewelina Namiecińska [1,], Magdalena Grazul [2], Beata Sadowska [3], Marzena Więckowska-Szakiel [3], Paweł Hikisz [4], Beata Pasternak [5] and Elzbieta Budzisz [1,*]

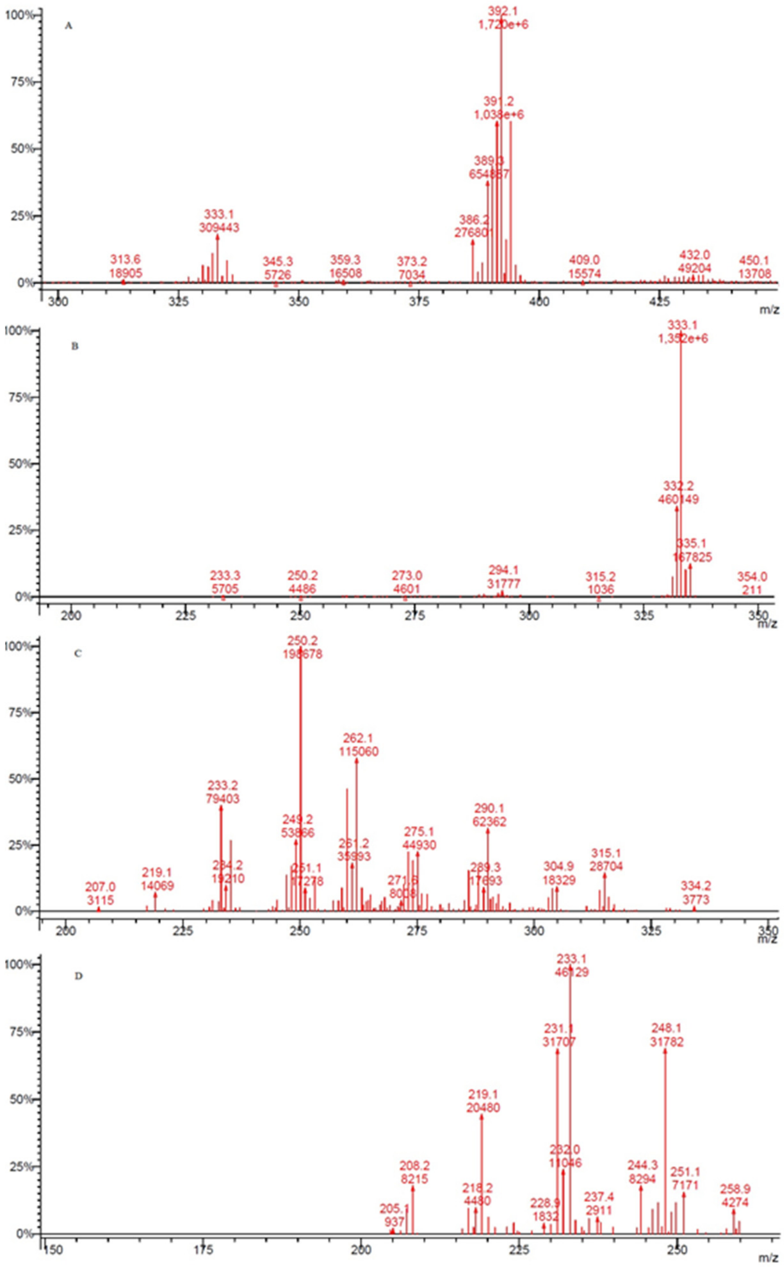

Figure S1. *ESI tandem mass spectra of compound* ***3d*** *in positive ion mode: A: ESI – full of compound* ***3d****; B: ESI-MS/MS of ion at m/z 390; C: ESI-MS3 of product ion at m/z 333; D: ESI-MS4 of product ion at m/z 250.*

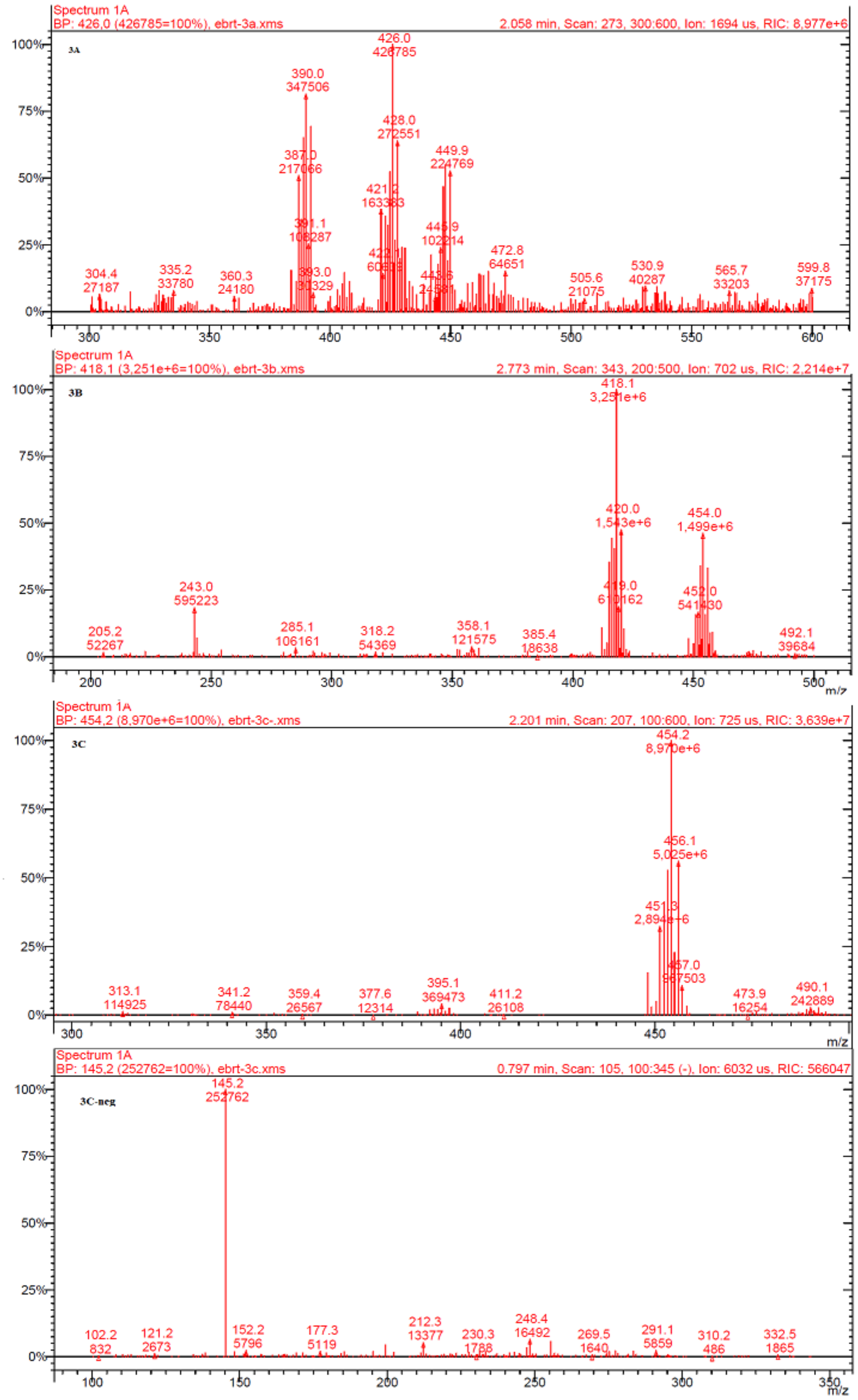

Figure S2. *ESI tandem mass spectra of compound* ***3a-c*** *in positive ion mode: A: ESI – full of compound* ***3a****; B: ESI – full of compound* ***3b****; C: ESI – full of compound* ***3c****; D: ESI – full of compound* ***3c*** *in negative ion mode.*

	S. aureus	*S. epidermidis*
**Control**	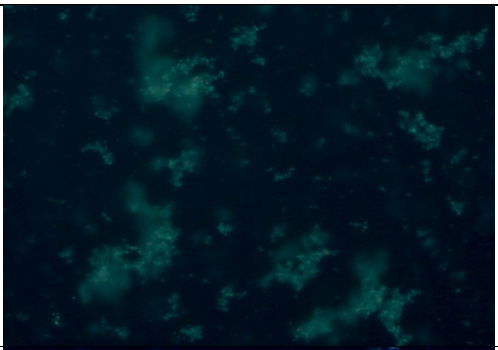	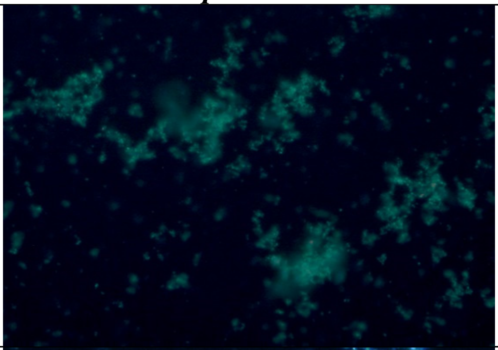
**2a**	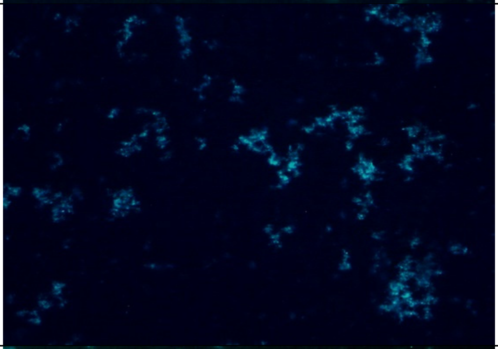	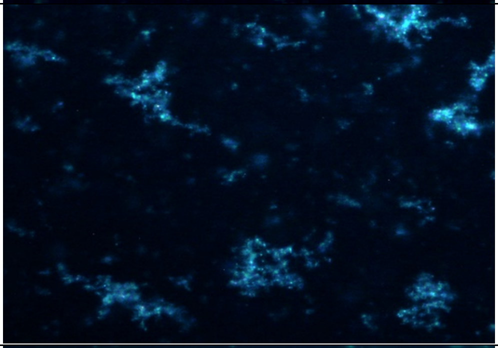
**2b**	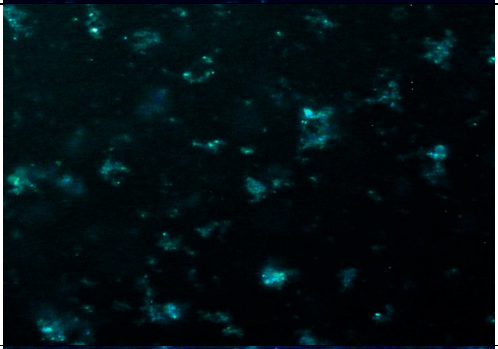	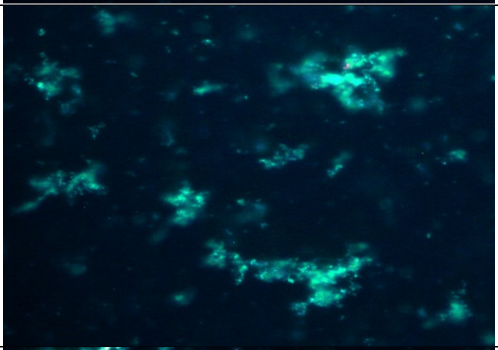
**2c**	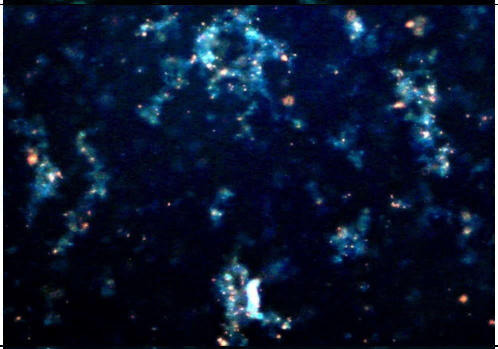	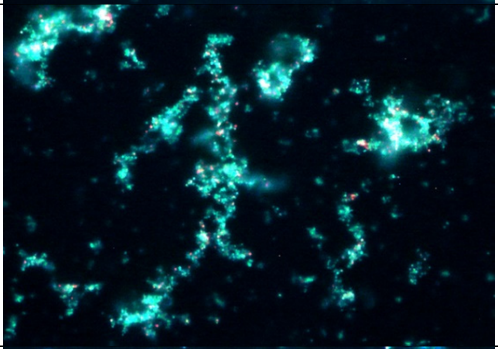
**2d**	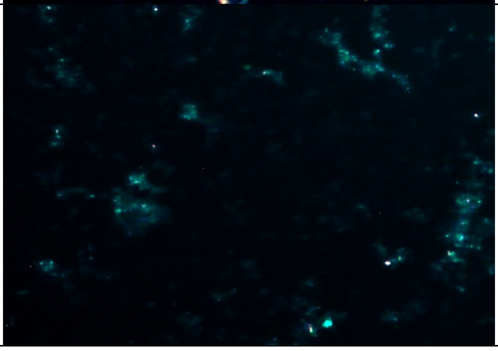	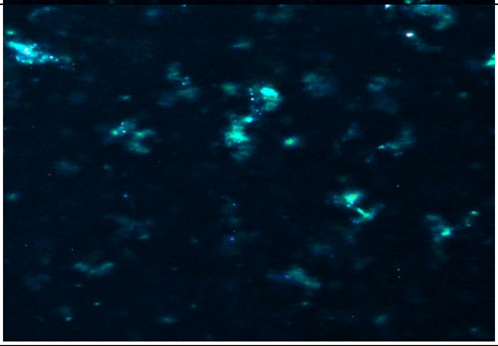

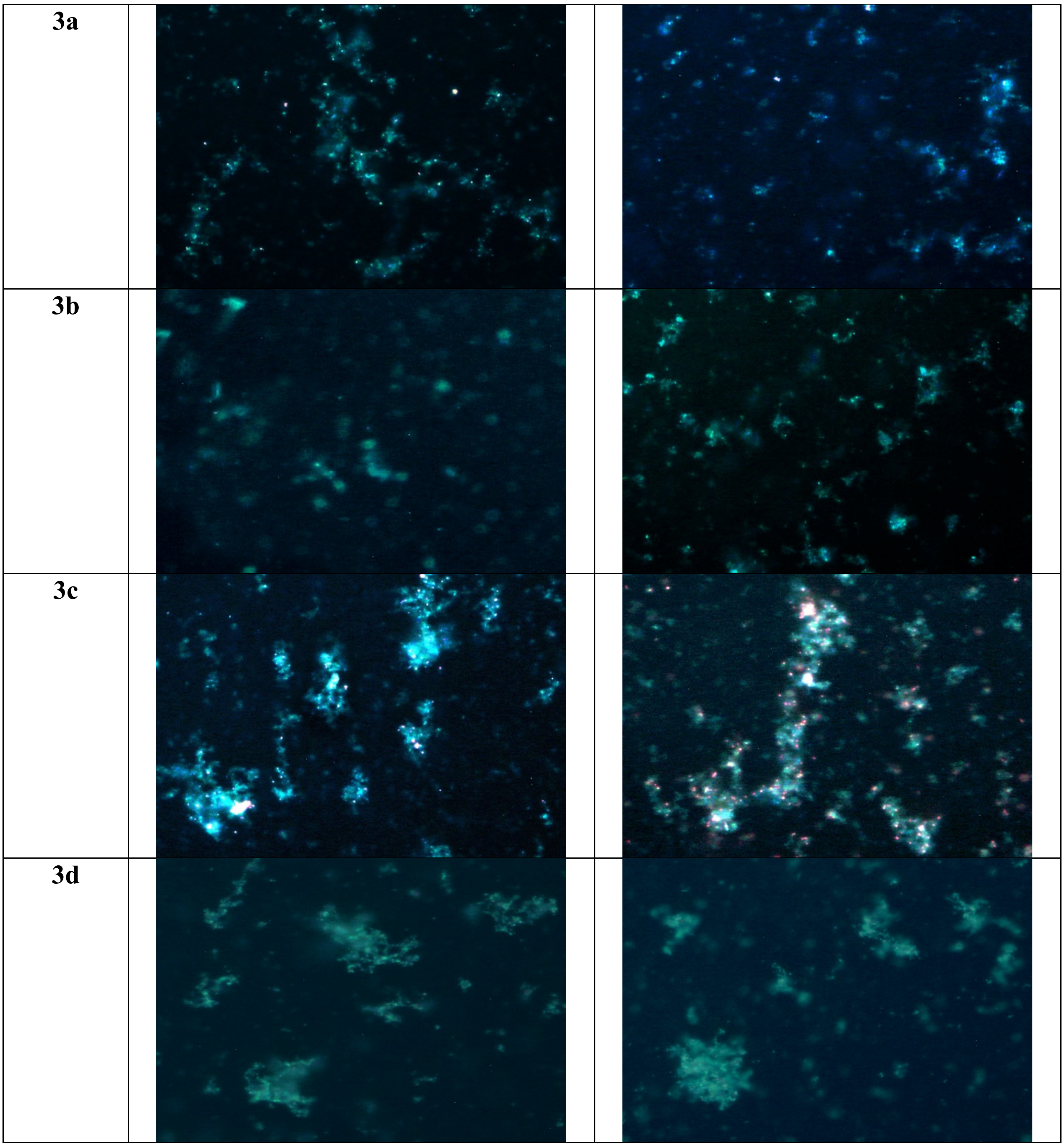

Figure S3. *Analysis the physiological state of bacterial cells stained with two fluorescent dyes - Hoechst 33258 and propidium iodide. Because of different bacterial cell membrane permeability living cells express pale blue fluorescence, while dead cells bright blue and red fluorescence.*

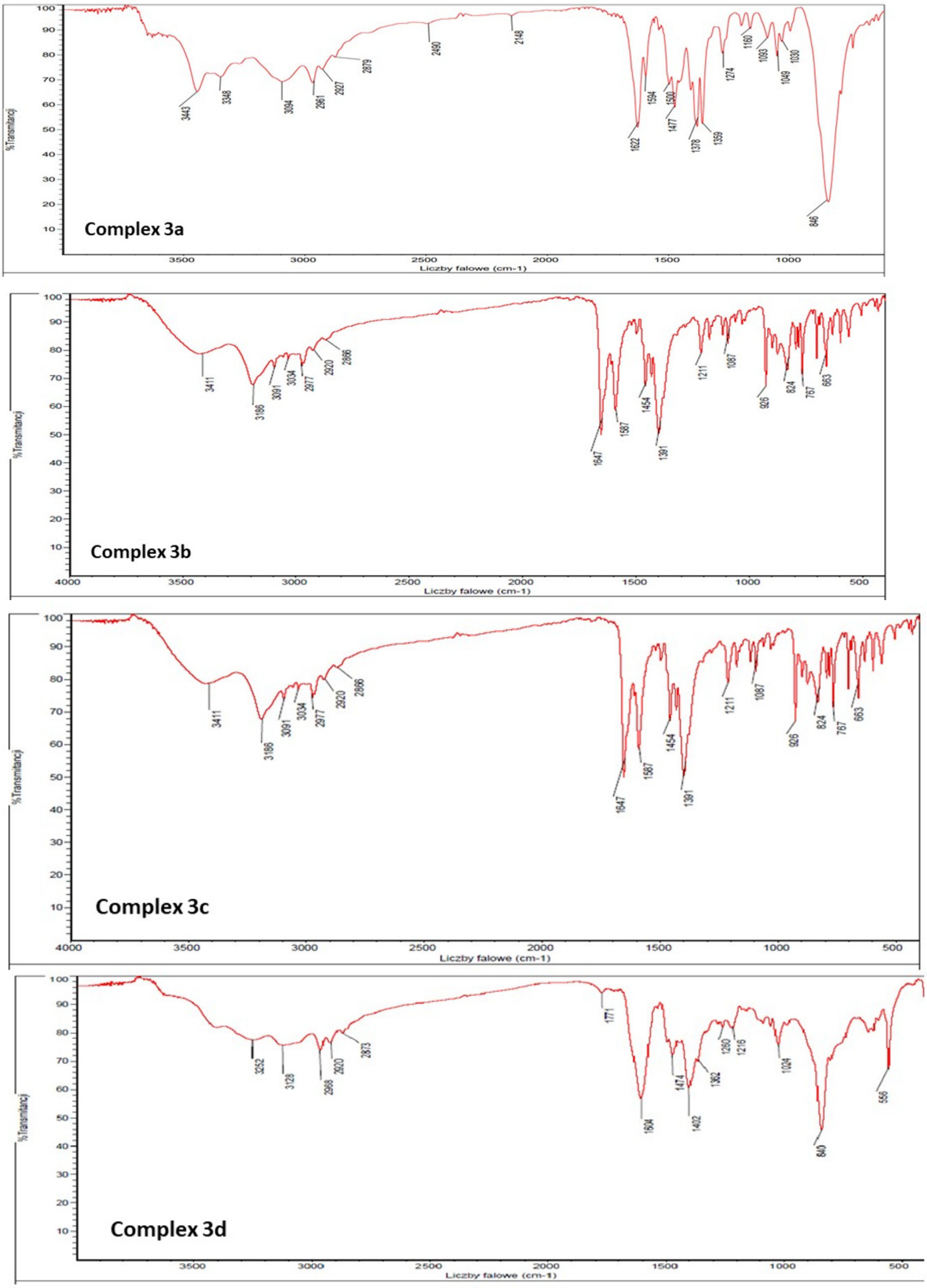

Figure S4. *FT-IR spectra for all complexes **3a**-**3d** (KBr cm^{-1}).*

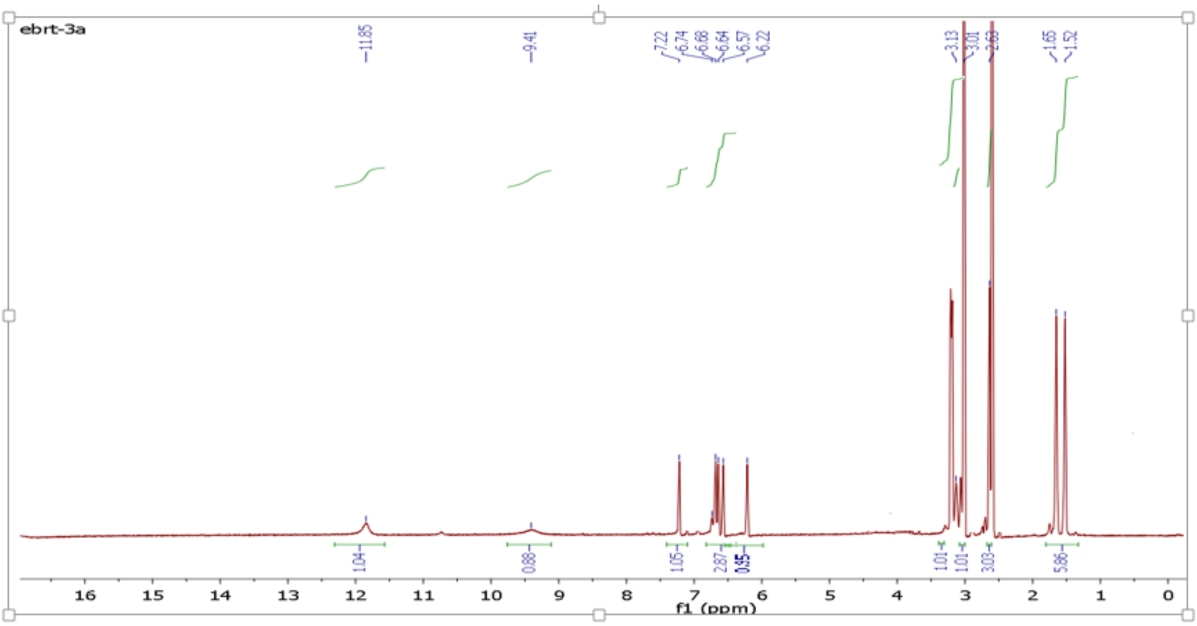

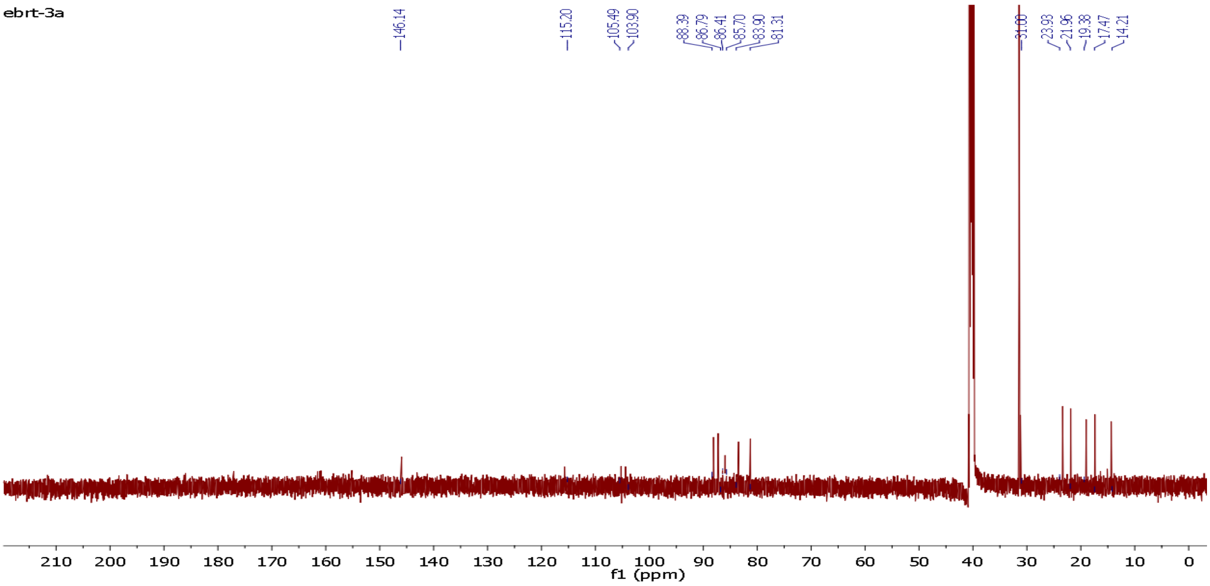

Figure S5. *NMR spectrum for complexes **3a** in $(CD_3)_2SO$-d_6.*

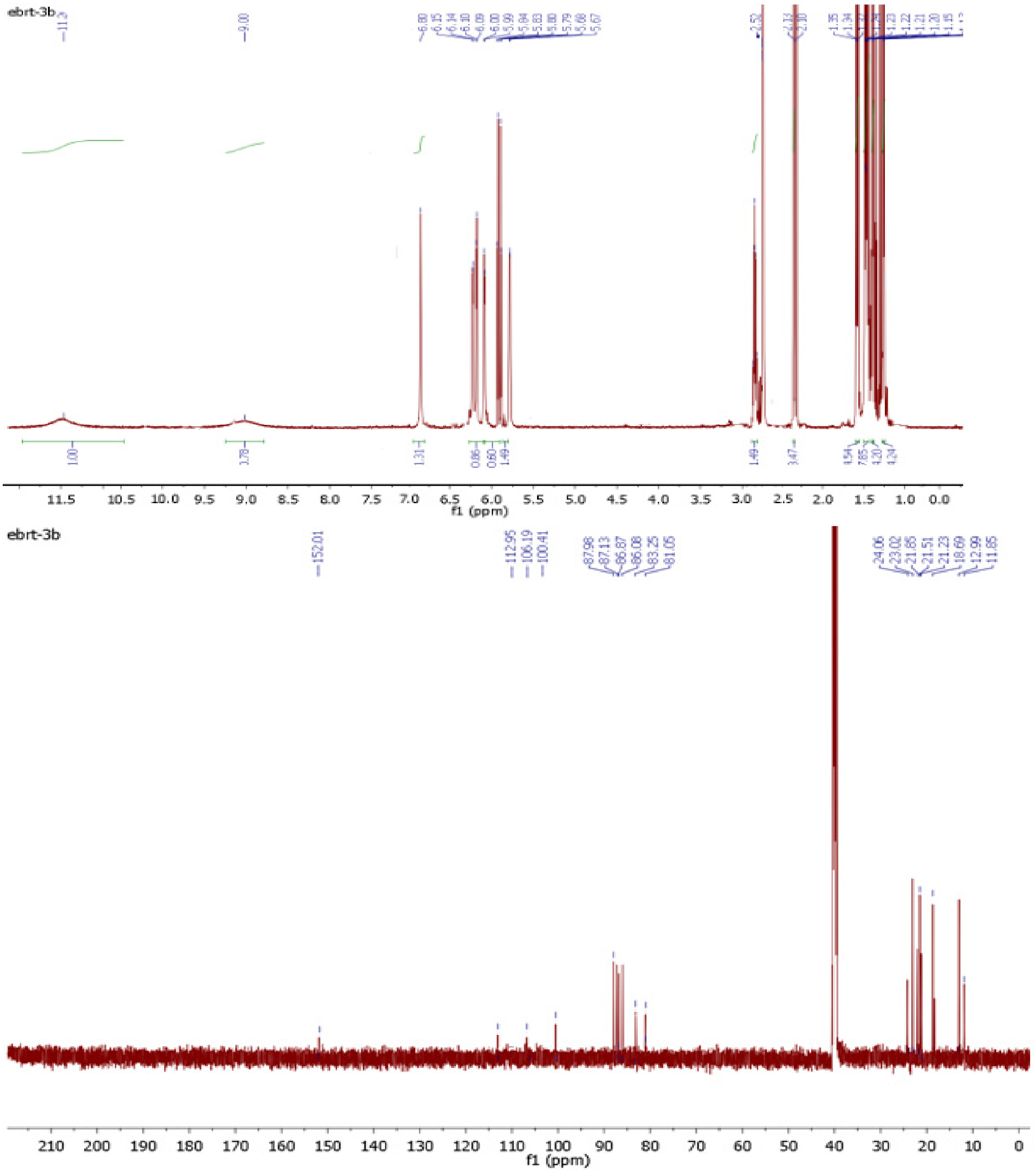

Figure S6. *NMR spectrum for complexes **3b** in $(CD_3)_2SO$-d_6.*

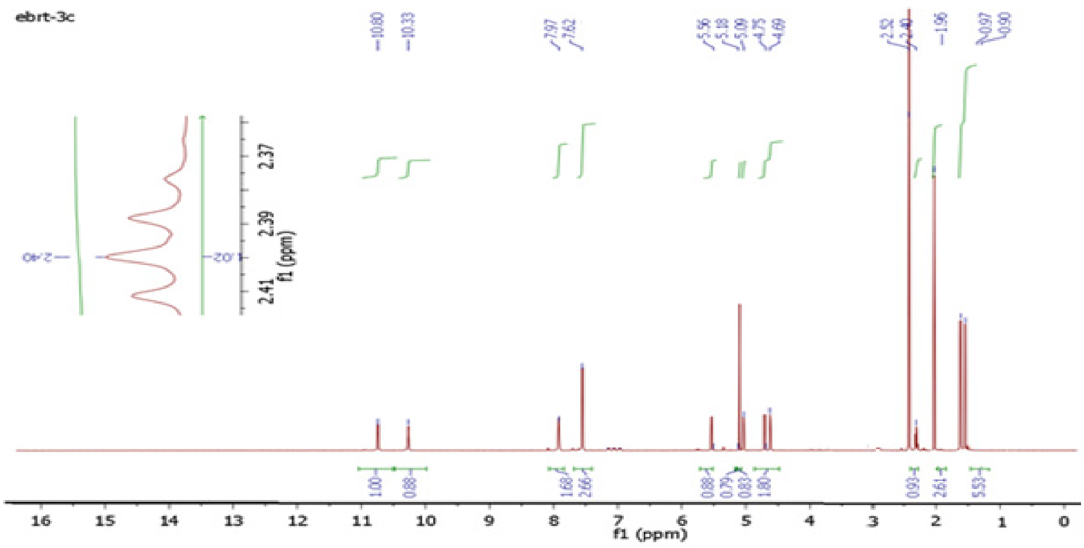

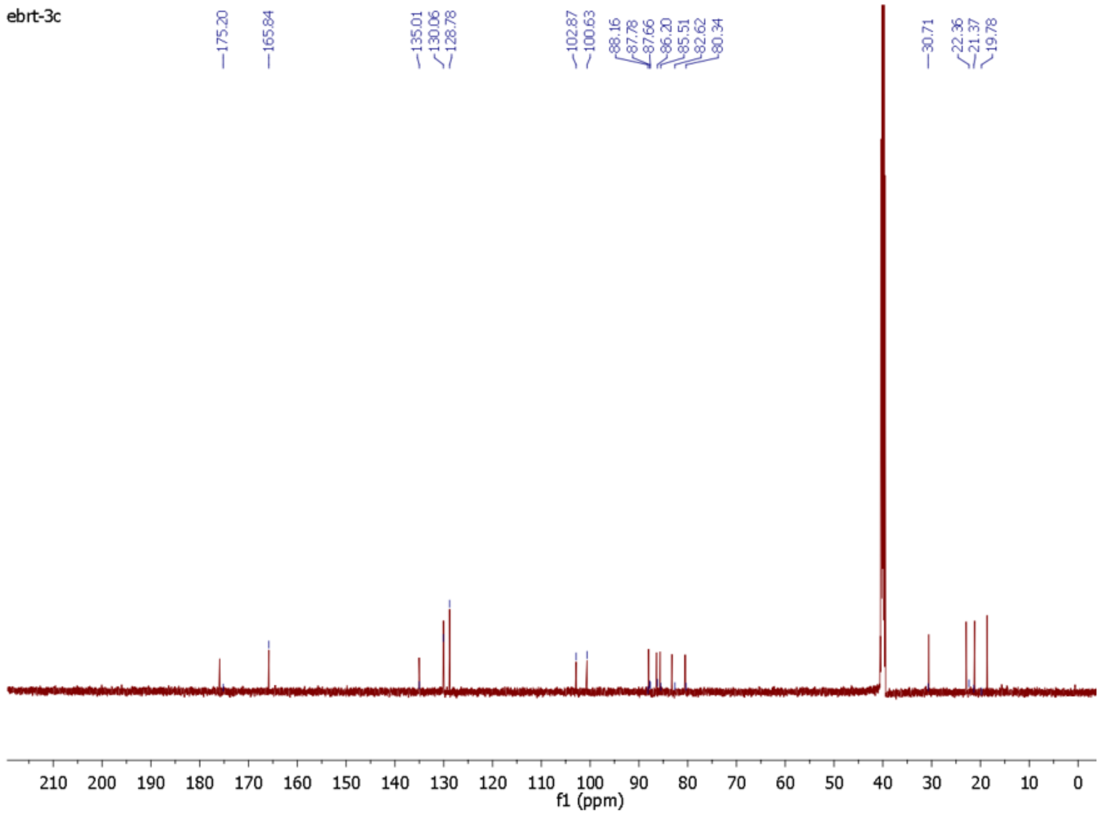

Figure S7. *NMR spectrum for complexes* **3c** *in* $(CD_3)_2SO$-d_6.

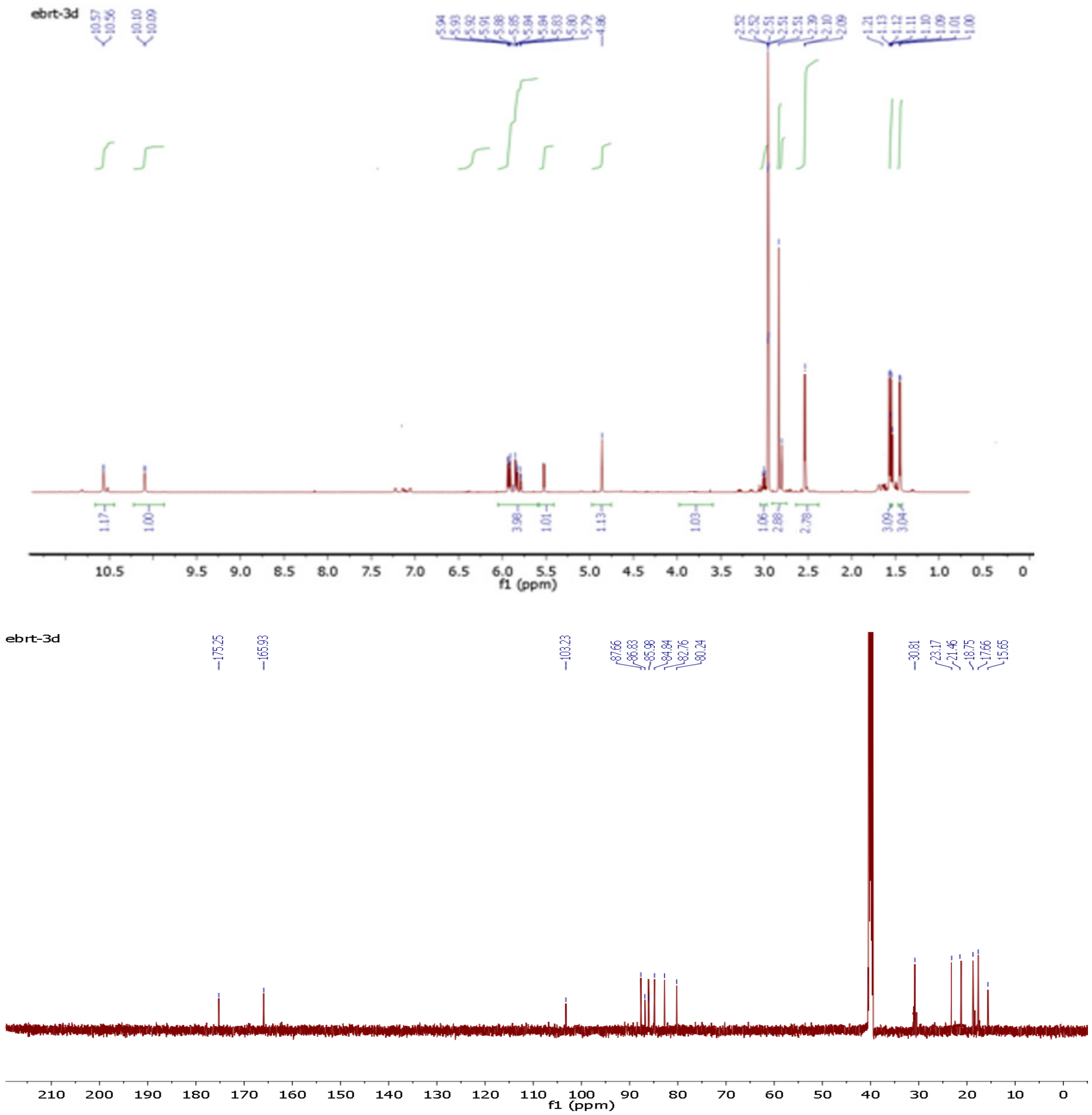

Figure S8. *NMR spectrum for complexes* **3d** *in* $(CD_3)_2SO$-d_6.

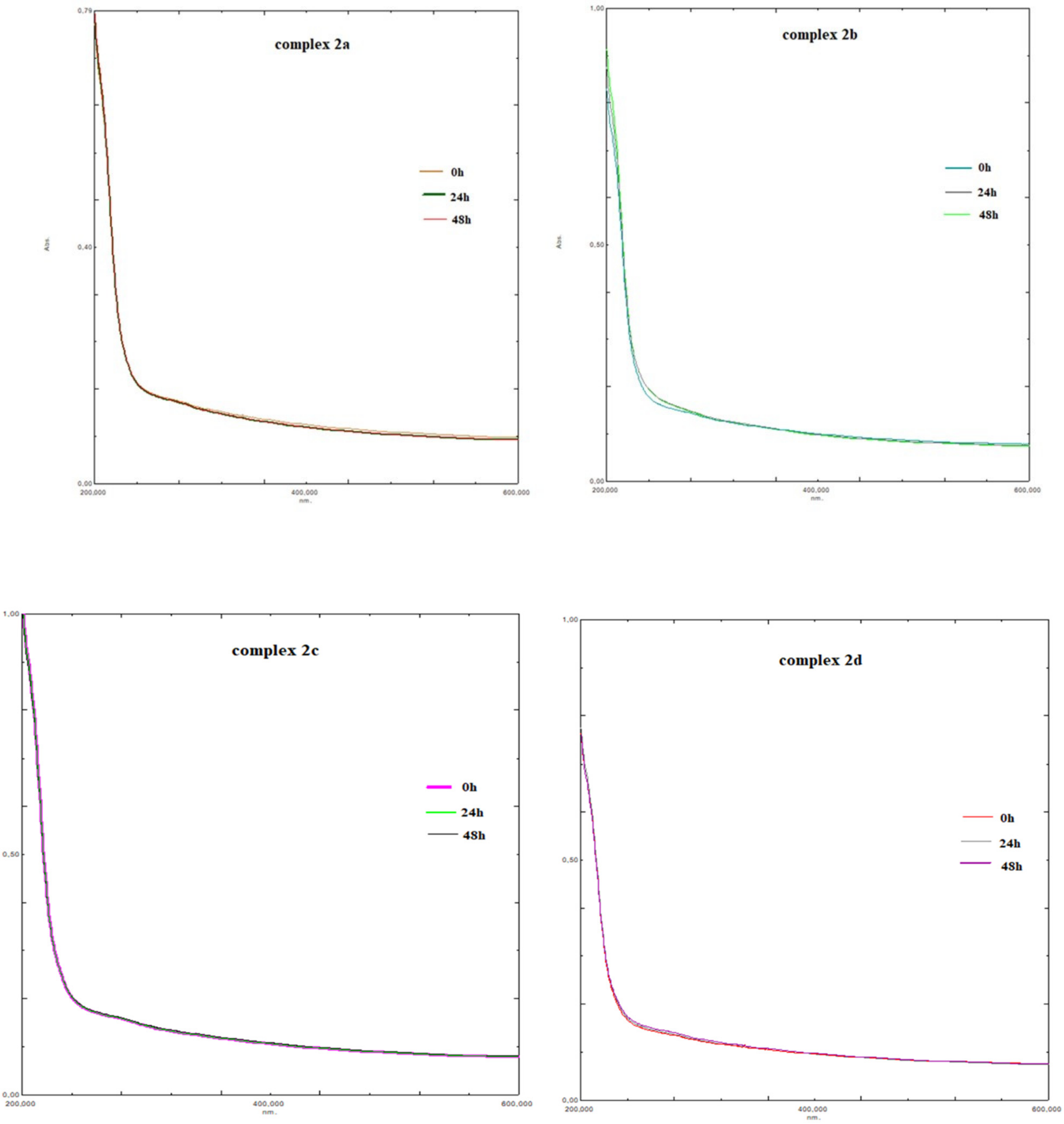

Figure S9. *The UV-vis absorption spectra of complexes* ***2a*****-*****2d*** *in (0,1%DMSO/water) solutions; concentration compounds* ***3a*****-*****3d*** *(1×10-9 M);the time experiment: after 0h, 24h and 48h.*

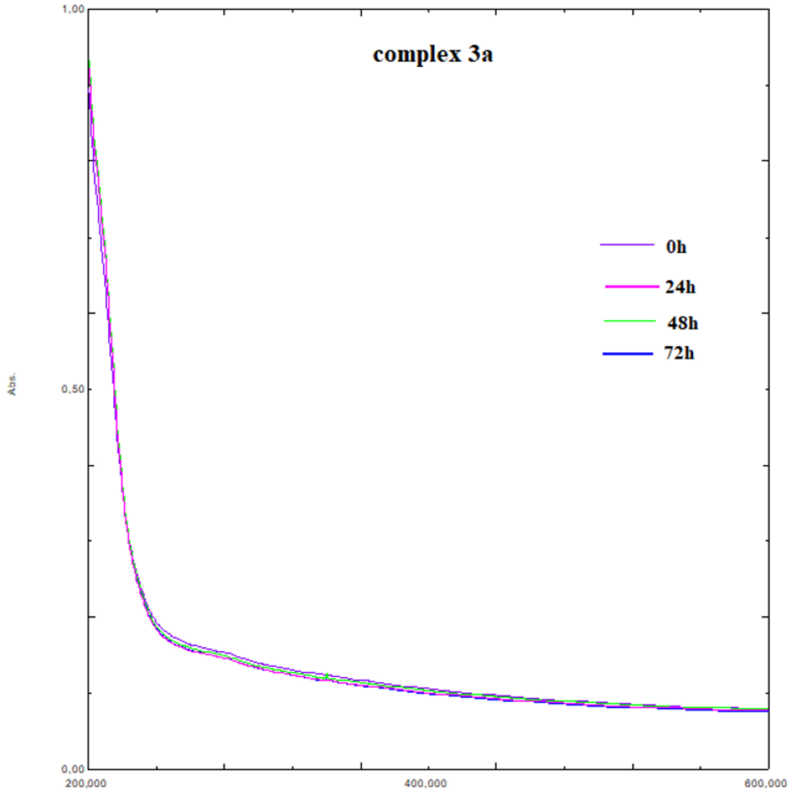
complex 3a
0h
24h
48h
72h
Abs.
1,00
0,50
0,00
200,000
400,000
600,000

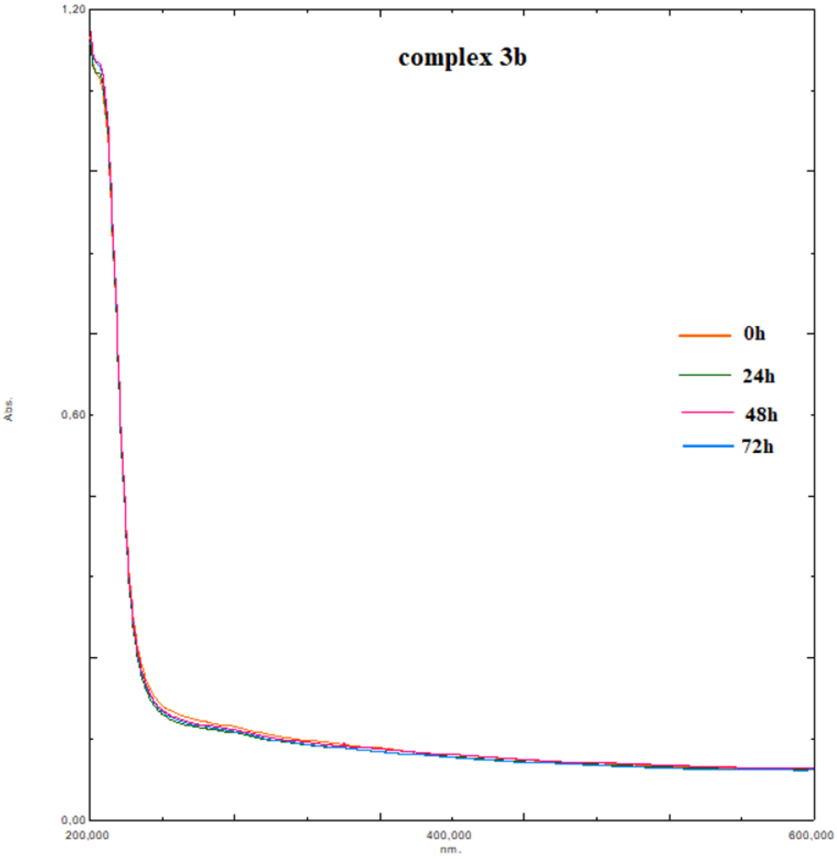
complex 3b
0h
24h
48h
72h
Abs.
1,20
0,60
0,00
200,000
400,000
600,000
nm.

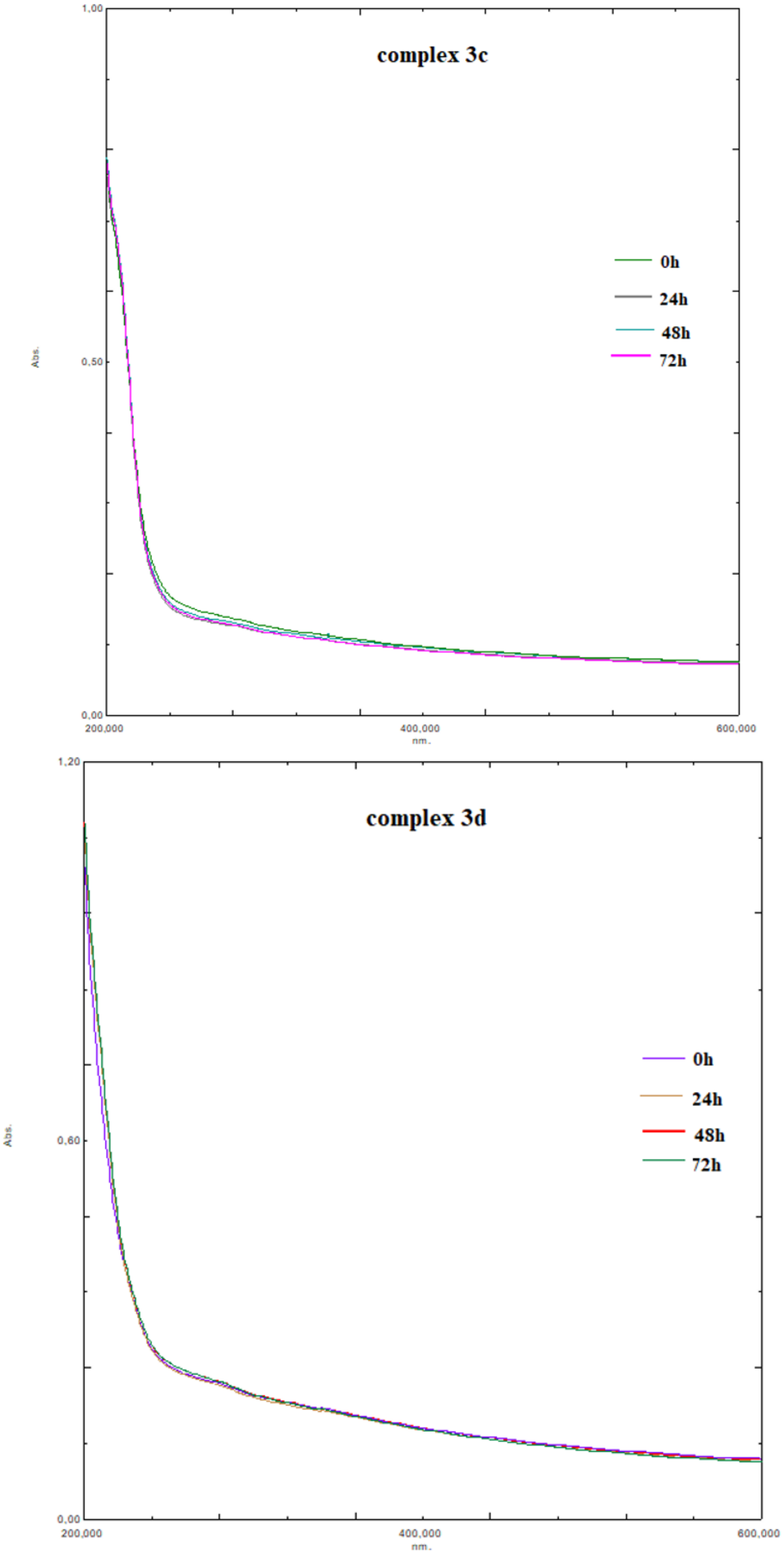

Figure S10. *The UV-vis absorption spectra of complexes **3a**-**3d** in (0,1%DMSO/water) solutions; concentration compounds **3a**, **3c**, **3d** (1×10-9 M) and **3b** (1×10-8 M); the time experiment: after 0h, 24h and 48h.*
